# Developing a blockchain-based digitally secured model for the educational sector in Saudi Arabia toward digital transformation

**DOI:** 10.7717/peerj-cs.1120

**Published:** 2022-09-29

**Authors:** Someah Alangari, Saeed M. Alshahrani, Nayyar Ahmed Khan, Abdulrahman Abdullah Alghamdi, Jameel Almalki, Waleed Al Shehri

**Affiliations:** 1 Department of Computer Science, College of Science and Humanities Dawadmi, Shaqra University, Shaqra, Riyadh, Saudi Arabia; 2 Department of Computer Science, College of Computing and Information Technology, Shaqra University, Shaqra, Riyadh, Saudi Arabia; 3 Department of Computer Science, College of Computer in Al-Lith, Umm Al-Qura University, Makkah, Makkah, Saudi Arabia

**Keywords:** Blockchain, Digital Transformation, Educational Sector, Saudi Arabia

## Abstract

New universities and educational organizations are increasing in Saudi Arabia with the increase in the need for high-quality education. This increased the need for a fast transformation to digitise the educational system in Saudi Arabia, which is one of the important pillars of the Saudi Vision 2030. The students who study in these organizations suffer the verification of academic records and other educational documents. Students who want to study at universities abroad also face the challenge of academic records and certificates verification. A secure, fast, and transparent model is required in the education sector in order to verify academic certificates issued by various educational organizations. Blockchain technology can be used with high data security to empower the educational sector of Saudi Arabia in the digital transformation and to help the educational organizations in verifying academic documents. In order to avoid any document fraud and forgery, along with the ease of verification of academic records and educational documents for the students. This research focuses on developing a model which will be helpful in achieving digital transformation in academic document verification by blockchain technology.

## Introduction

The verification of transcripts is one of the most tedious and time-consuming tasks. The education system of Saudi Arabia, comprises of the Ministry of Education on the top. This is followed by various universities, which are public and private in the kingdom. Primary and secondary schools are also a part of the Ministry of Education umbrella. It is usually observed that whenever a student in the kingdom tries to connect and attest a degree or document from the Ministry of Education is a transcript, has to go towards a sequence of steps. The commission of attestation, takes some time to approve the validity of transcript of a student. Amid the COVID-19 pandemic, various sectors suffered a lot of problems related to the execution. One of the important sectors is the educational sector, which suffered the maximum globally. An alternative solution for providing digital transformation and its allied services across organizations imparting education is expected. As a use case, if we assume that the student is applying for higher studies in a country outside the kingdom, is supposed to verify his documents and submitted to the Embassy of the other country along with the University, in which he is applying for higher education. The process of verification and attestation of the transcript, comprises of a number of stakeholders which participate starting from the Ministry of Education till the University in which the student is applying. The blockchain technology since the recent past emerged as a game changer that can help the Ministry of Education and universities to collaborate collectively towards the verification of documents that can be used by the students to apply in the form of digital transcripts (Murugesan & Benakanahally Lakshminarasaiah, 2021).

Blockchain is one of the core technologies, which is responsible towards the creation of crypto-currencies, commerce systems, judiciary systems and various finance-based projects. A large number of applications in educational units let students face various problems. Blockchain can find solution to these problems persisting in the academic sector. Vision 2030 of Saudi Arabia focuses on implementation of digital transformation across various sectors in the country including educational sector as well (My.GOV.SA, 2022). The digital transformation will be helpful enough to facilitate the citizens and residents for enjoying quality living. Blockchain is supposed to be decentralized, straightforward, as well as secure to provide solutions towards problems like absence of trust, higher cost of execution, security, authenticity and global network. This research, which is centralized around academic sector, focus mainly upon the implementation of blockchain-based model in the academic domain to facilitate students, universities, educational organizations and recruiters with a no hassle blockchain network verification system. With the development of information technology and services, digital transformation is experienced in various sectors across the world.

Blockchain has emerged as one of the most important and challenging technology in the recent past. Chen et al. (2018) provided a large number of applications that can be integrated in educational sector as an application of blockchain technology. Certain benefits and challenges were also explained towards the implementation of these services. Furthermore, Bhaskar, Tiwari & Joshi (2020) suggested an analysis towards the barriers and benefits of blockchain in education system. With the increase in desire towards learning across the kingdom of Saudi Arabia and its citizens, large number of educational organizations are arising. However, all the organizations are not accredited or are still in the process of accreditation. The surveys and analysis given by Fedorova & Skobleva (2020) raises our attention towards the applicability of blockchain in educational sector. Any type of forgery or fraud is impossible, which contributes towards the integrity and security of the documents and the certificates marked as blocks in the chain. The data records are preserved with a high level of security and latest technology (Baker, Nikbakht & Smith, 2021). The hash encrypted blocks available inside the blockchain supports a smart contract which is fault-tolerant and effective. The processing of the transactions is very smooth, which promises low latency and confidentiality in the process of transcript verification round the globe for all the Saudi Arabian students and universities. The main idea and motivation behind this research are to facilitate the educational environment with the ease of transcript verification in a global scenario (Bhaskar, Tiwari & Joshi, 2020).

A conceptual progress is taking place with the help of blockchain technology to be integrated in educational sector providing security of data, privacy, effective and trustworthy system (Raimundo & Rosário, 2021). An interesting implementation of blockchain technology proposed by Turkanović et al. (2018) attracts the audience with its global acceptance and ubiquitous environment of edge execution. Dash et al. (2022) give a detailed description about its applicability across government organizations. Various benefits and obstacles were discussed in this study. Kumutha & Jayalakshmi (2022) give a critical review across the certificate authentication and verification process that can be regulated with the help of blockchain. The authors raised authenticity system in order to avoid fraud and forgery in educational documents (Andoni et al., 2019). With the help of such innovative ideas, this research focus mainly upon proposing a blockchain driven technology oriented digital system responsible towards providing the educational sector brilliant digital transformation in the kingdom.

In various countries it has been observed that document forgery and illegal use of fake transcripts has been reported (Ghaffar & Hussain, 2019). The Ministry of Education and the universities of the kingdom of Saudi Arabia focus on digitization of information under the Vision 2030 of the Saudi kingdom. To facilitate this, use of blockchain technology creates a robust, has enabled, decentralized and secure mechanism to store the records of student’s transcripts on the network (Kumaresh, 2021).The following objectives are expected to be achieved with the prescribed model in this research:

• Identify and study various online transcript verification systems which are traditionally centralized database oriented
• Propose a new novel platform, which is capable enough to authenticate transcripts of Saudi Arabian students on a global scenario.
• The architecture proposed in this paper makes use of hyperledger fabric blockchain to achieve the desired goal in a secure and transparent way (Dhillon, Metcalf & Hooper, 2017).
• The activities, as well as the operations are illustrated in the form of sequences to represent how the blockchain will propagate further to create a transcript verification process.

In this article, we discussed the blockchain technology in the first half. The next section focuses on showcasing the proposed model, which makes use of hyperledger fabric technology using the distributed application framework (DAPP) (Mishra et al., 2020). The implementation of the proposed solution and challengers are discussed in the last section.

## Background

Blockchain is defined as a chain of blocks which is connected together an associative with each other in the form of a distributed ledger that is accessible from all the peer network. Earlier it was used only to store digital currencies and transactions (Swan, 2015). However, more bright and effective utilization of blockchain were done in various systems that require concurrency and digitization. Public blockchain, private blockchain, and consortium blockchain are three major categories for such network of digital blocks. It is a revolutionary technology, which is impacting modern day to day life with the help of providing security, transparency, as well as decentralization of data in the form of blocks.

### Primary Concepts

• P2P Network: A distributed network which is having an architecture to share information amongst various partner pears is called as P2P network (Schollmeier, 2001). All the partners in the network, create a pool of resources including various computational hardware as well as software units accessible by all the peers. All the nodes act as client as well as servers. On demand availability of resources is maintained in such a network that promises high availability.
• Cryptography: The art of hiding the information and making secure communication in digital data can be called as cryptography. Symmetric and a symmetric key technique are used to bind the data in order to maintain its confidentiality and availability to avoid security breaches (Schneier, 2007). Authentication and data access are two important process involved in this encryption and decryption phase. Public key cryptography and its applications are widely used across various web-based communication systems.
• Hash Chain: To preserve the integrity of data, hash value, for the data block is calculated with the help of mathematical functions. These functions are called as hashes. The hash value generated with the help of such function remains unaltered and irreversible. Hash chain comprises of the hash value calculated for various blocks and collectively decrypted at the destination (Lamport, 1981). It becomes really difficult to compute all original data block from the hash values which provides data integrity and plays a very important role in blockchain.
• Merkle Tree: Merkle tree is a hash tree which is used to arrange data and corresponding hash values in the form of a tree (Merkle, 1982). Every leaf in the tree, comprises of the hash for data block, which in return creates a bigger hash for the leaves at a particular level. The top value comprises of the hash for all the child in the tree. Every parent node contains the hash of data hold by the child nodes. This creates a very strong encryption unit, which becomes the backbone of a blockchain.
• Digital Signatures: Digital signatures provides a complete means of authorship for the contents that are signed with the help of the signature. It assures that the information is not forged, unused, and non-repudiated (Esposito et al., 2018). These digital signatures cannot be shifted on another documents or data packets. When timestamp is integrated along with the digital signatures the tracking of data becomes very powerful, which is used in blockchain transactions.

### Blockchain characteristics

Blockchain characteristics: various characteristics of blockchain are identified in different researches. Since the technology is in its infant stages, it is probably likely that various issues and features can evolve eventually. Decentralization is one of the most important characteristic of blockchain (Xie et al., 2019). In the network that works as a P2P network, data blocks are managed in a decentralized way. All the nodes are capable of updating the data packets. However, consensus protocols are applied to authenticate the integrity of transaction.

Multiple copies of the transaction are reported on the blockchain network due to these protocols. Also, the removal of information from any of the data package in the block reports to update of information across all the nodes in the P2P network. High level of accountability is given in blockchain networks with the help of transparency (Francisco & Swanson, 2018). Several validations are done at node level iteratively. Availability of public addresses of the nodes along with the transaction records leads to a strong technical foundation in blockchain, which provides tracking and verification of information. Individuals can find out a strong range of personal information from any block available in the nearest peer. Smart contracts are used in blockchain 3.0, in which trusted administrator acts as third-party to avoid any kind of data manipulation issues (Nugent, Upton & Cimpoesu, 2016).

The transactions in a blockchain network takes place with the applicability of trust. All the parties involved in the transactions in a blockchain makes use of cryptography and mathematical hash functions to prove the authenticity of the data. Consensus protocols and algorithms updates the distributed ledger of blockchain to solve the problem of transactions and maintaining system integrity (Sompolinsky & Zohar, 2015). Middle man from all the transactions are removed in a very secure manner (TC, 2017). The security keys in the blockchain system approves the authentic nature and confidentiality of the transactions in the P2P network. Immutability, accounts for the un-tamper ability of the blockchain (Dunjic, 2018). It means that once the data is entered into the blockchain, it will remain unaltered and cannot be deleted or changed in any case (Esposito et al., 2018). For financial transactions and audits this feature of immutability is highly recommended and appreciated by large number of clients using blockchain networks (Lu, 2018).

The traceability to identify the domain, including source and destination of a data block in the P2P network is yet another powerful characteristic of blockchain (Rejeb, Keogh & Treiblmaier, 2019). Data integrity at very high level of trust is maintained. Time stamping and hash code for every data packet or block makes it easily traceable in the Merkle tree. Anonymous nature of uploading the data in the block retains the privacy of a user and avoids any type of unauthorized supervision or stalking (Alsaqqa & Almajali, 2020). The exchange of data between nodes takes place with the trust parameter and done anonymously. All the users in the blockchain network can connect to others with their identities hidden. All the decisions that takes place in such a P2P network, assures a democratic way of working (Zheng et al., 2018). The shared ledger in the blockchain is updated with an immediate effect in which all the nodes practically takes participation. Equal rights to share data, update information and request is provided to all the nodes in the chain (Baliga, 2017). The integrity of the system is never compromised and the data remains accurate and consistent in the entire lifetime. Multiple copies of the data reside at various P2P nodes which guarantees the reliability and integrity to a larger scale (Baliga, 2017).

### Challenges and issues

Despite of the first momentum that blockchain technology has acquired various issues remains unanswered. Scalability is one of the major factors which leads to the limitations of high latency and resource requirements in blockchain. Because of this various performance issues are observed in such kind of P2P network (Yang et al., 2019). Cluster architecture and performance evaluation of blockchain is still awaited at a larger scale (Wang et al., 2019a). Even the cost of decentralization for blockchain networks is hard as compared to general networking topologies. The financial obligations for using public blockchain is a big challenge towards the open source community (Wang et al., 2019a).

The immutable nature of the blockchain may result in bugs which are reported at the time of deployment in smart contracts. These irreversible bugs need to be checked or updated depending upon the nature of the blockchain (Wang et al., 2019b). As per the basic principle of design of a blockchain, no data packet can be modified or altered by anyone. So, it becomes really difficult to handle the problem of immutable smart contract bugs (Francisco & Swanson, 2018). Energy efficiency of the network, attack on data integrity of the blockchain, centralization issues, immutable hindrances are some of the important underlying factors which needs to be solved (Kiayias et al., 2017).

### Architecture of blockchain

Generally, the blockchain is used to store financial transactions in an entity called a block. These blocks are restricted towards any changes and updates. Every block, comprises of a header and a block body as a part of the data structure. The capacity of the block is defined by the size in the blockchain. Full nodes and lightweight nodes are generated comprising of several blocks. If or not can work as a server having the capability of updating the history and adding data in the node. Several other types of nodes such as archival nodes, miner nodes, staking nodes, authority node, Master nodes, lightweight node and pruned nodes are also a part of a blockchain. All these elements make blockchain 1.0 as the first of its kind (Gruber, Li & Karame, 2018).

Blockchain 2.0 also referred as smart contracts became popular due to its capability of enforcing agreements in a financial transaction between two parties without the requirement of trusted third-party (Lande & Zunino, 2018). Ethereum and Hyperledger are some open source platforms that can be implanted as blockchain. Blockchain 3.0 is the new version, which is implanted. Not only with financial transactions, but also to other industries which involves distributed applications. The use of Internet of things and programmable hardware was done in this architecture of the blockchain (Zhang et al., 2021). Various level applications are prescribed that makes use of such a technique. Various applications run at different layers of architecture in the blockchain. Application layer, contact layer, incentive layer, consensus layer, network layer, and data layer comprise of a layered architecture for blockchain applications. Various frameworks like Bitcoin, Ethereum, Hyperledger, Tron, Multichain, Openchain, Quorium, Iota and Exonum are proposed as Consensus mechanisms globally (Sankar, Sindhu & Sethumadhavan, 2017). There are various open source and commercial application development frameworks that are used as blockchain for various applications.

### Security in blockchain

Despite of the various security issues which are mentioned, advanced cryptography primitive algorithms are used in blockchain network to ensure the security of the information. The use of mathematical hash functions such as SHA256, RIPEMD, PNG, MD5 etc. Salman et al. (2018) enables the blockchain to be safe and authentic. Digital signatures created by various well-known algorithms like RSA or Euclidean mathematics ensures the safety of the public key encryption system. The very well-known DSA algorithm ensures that the signature of the block remains legitimate in nature. ZCASH or zk-SNARK algorithms are very well-known for financial transactions and their safety. They are integrated in the blockchain providing extraordinary performance of the P2P network. Monero Ring Signature or Zero Knowledge Proofs are also some of the mechanisms which proves the safety and authenticity of the blockchain (Christidis & Devetsikiotis, 2016), thereby resulting in the integrity of the data block, which is saved inside the blockchain.

## Proposed Model

Ministry of Education Transcript Verification blockchain (Saudi Arabia) (MOETVBC)—The degrees issued by the universities and higher education institutes are significantly used by various recruiters, recruitment agencies, multinational organizations, government department and universities abroad. Online learning was promoted during the global COVID-19 pandemic. The current academic system was shifted on digital media, including attestation of the documents (Caldarelli & Ellul, 2021).

In this research, we tried to propose a model, which works upon hyper ledger fabric blockchain for the transcript verification of Ministry of Education in Saudi Arabia. The documents and degrees, owned by various students are recruited by the universities in which the study. At the time of transcript verification, the University offices and the Ministry of Education have to perform a series of tasks to finally attest and send the final copy of the approved certificates. The model proposed in this study comprises of three main portions:

• Blockchain based Secure Record System for Ministry of Education
• Transcript verification blockchain using hyper ledger
• Digital record exchange network (P2P Network based on blockchain)

These phases are explained as per the working characteristics in the subsequent sections below. There are various techniques to achieve digitally these sections. But the most general and common techniques are illustrated. The platform which is expected in this case can make use of any technical solution needed to achieve them. However the use of Hyperledger is a must to generate a typical blockchain environment.

### Phase I for the model

**Figure 1:**
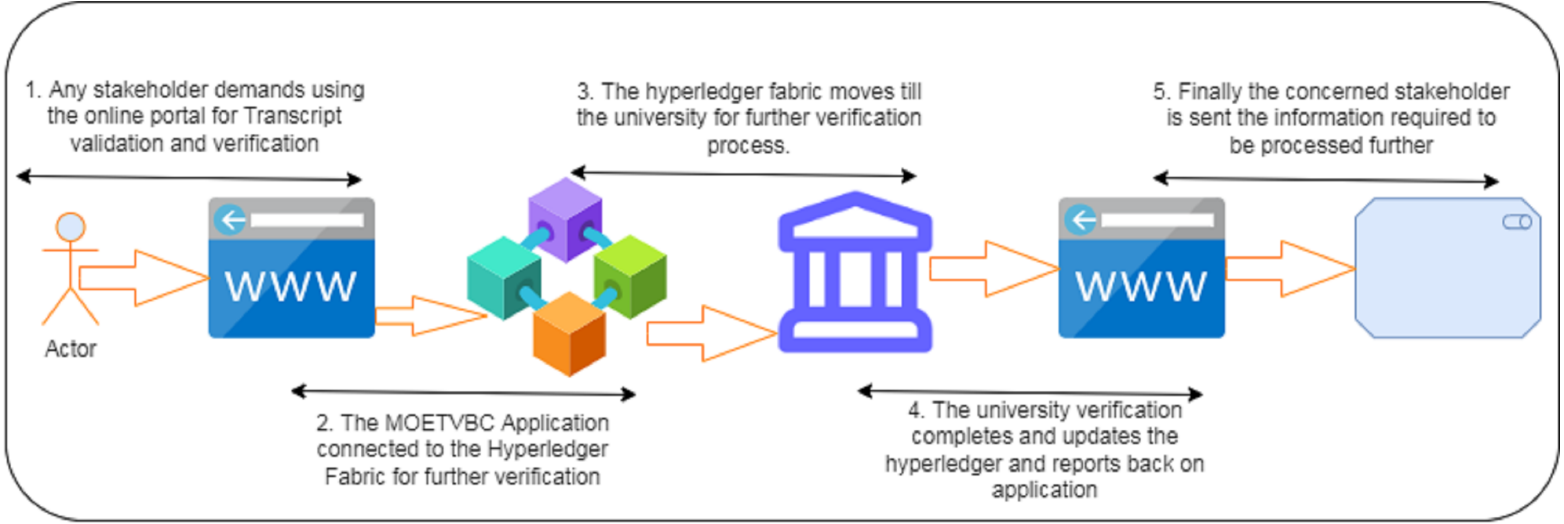
Phase I for the MOETVBC proposed model.

Phase I for the prescribed model is depicted in Fig. 1 above. On a macro level, the user who is trying to get the transcripts verify is able to see the portal/distributed application with the help of his authentic login. Once the login is verified his request is subsequently logged into the hyper Ledger fabric for further consideration by the authorized stakeholders. The verification of the transcripts takes place in association with the University/educational organization from which the applicant has completed his academics. Once the University verification is done, the results are updated on the portal. Phase I is the overview of the user sight for the transcript verification.

### Phase II for the model

**Figure 2:**
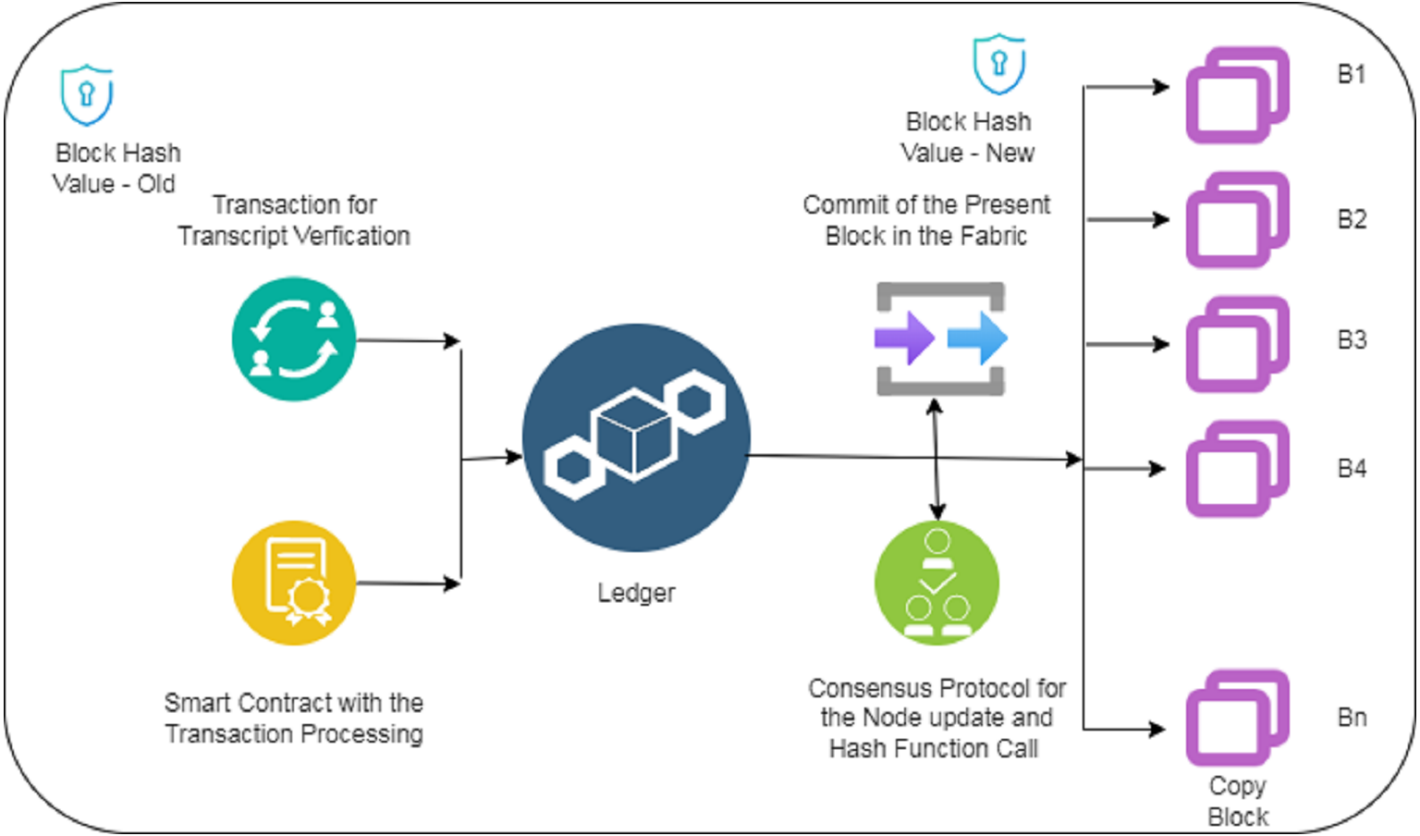
Phase II for the MOETVBC proposed model.

As shown in the Fig. 2 above is the second phase for the proposed model. This is supposed to be a very important part of the entire schema proposed. The block that is created from the applicant contains the block hash code. The information asked by the applicant goes as a transaction for transcript verification procedure. An immediate smart contract for the transaction processing is created based on the request of the applicant. All the information along with the hash code is logged into the ledger. The consensus protocols call the hash functions to check the authenticity of the block generated by the user. The commit for the information submitted by the applicant after the verification is done with the help of consensus protocols. Once the approval is done, the copy of the user information/block is submitted to various adjacent P2P network nodes (Vlachou et al., 2020). It should be noted that the final hash code for the block before sending it on all the nodes in the P2P network is also calculated and attached with the block, which contains the address of the present block.

The transaction information comprises of user credentials and information along with the hash of the previous block and the hash of the present block. Once the ledger commits the complete set of information, the final hash is calculated and appended with the hash address of the upcoming block (Lu et al., 2020). The security of the blockchain notes lies in the fact that all the blocks contains the hash value of the previous and upcoming node in the entire chain. Any changes/updates that might happen in any of the block will result in a change in the hash value of the blocks. The consensus protocols check and updates whether all the block hash values are appropriate and are not altered for any unethical access (Khan et al., 2021a). This makes the blockchain very powerful and transparent in terms of security.

### Phase III for the model

**Figure 3:**
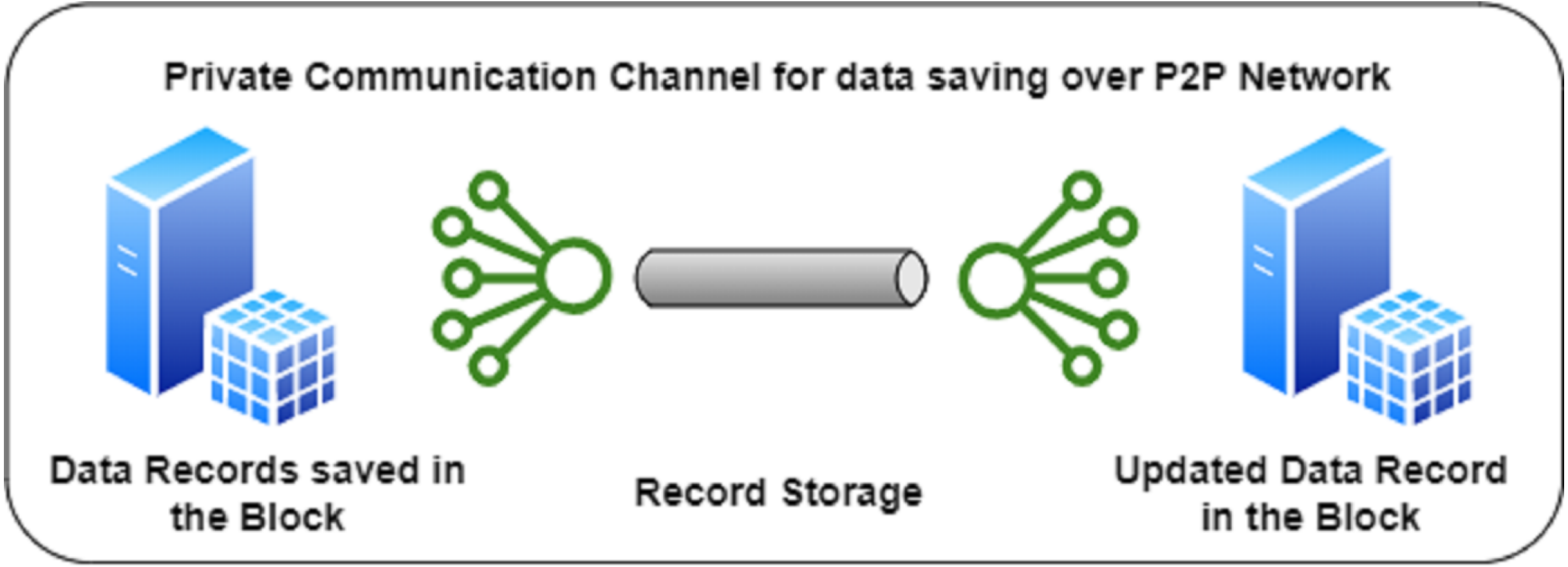
Phase III for the MOETVBC proposed model.

The final phase after committing the blocks in the ledger takes place by saving the records in the decentralized storage across the complete blockchain. Once the transcripts are verified by the verifying authorities/stakeholders, the results are sent back to the Ministry of Education and the complete block for the applicant’s transaction is fulfilled. Figure 3. above shows the schematic representation of the Phase III. The final block, comprises of all the contracts and certificates that are exchanged by various entities were verifying the transcripts. After all the verification is done, the hash value of the final block along with its data is updated in the record on the P2P network terminal nodes. The information is exchanged with the help of the decentralized application to the user. The sharing of verified transcripts by the user is now possible with the limited access to various organizations across the world.

### Working of the proposed model

**Figure 4:**
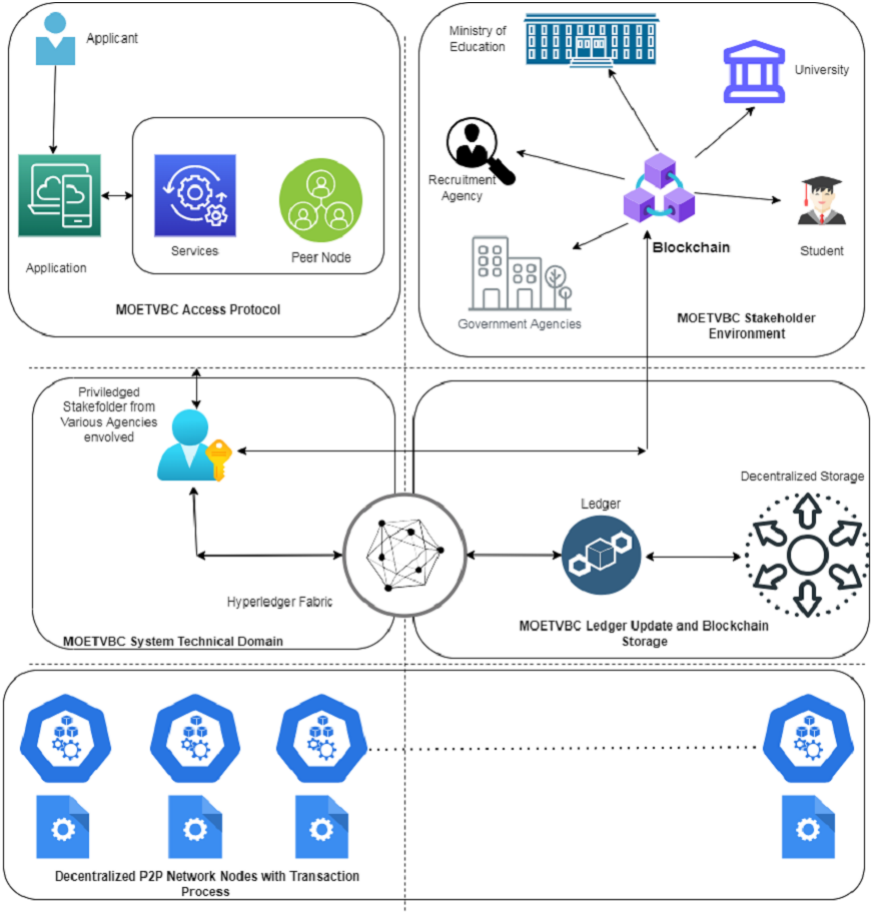
The MOETVBC proposed model.

Let us consider the Fig. 4 shown below to completely understand the working of the model. The Ministry of Education will deploy the initial ledger that will be used for storing the records in the beginning. Once a student/applicant applies for the transcript verification using the portal/distributed application the request goes to the concerned engineer. As soon as the request reaches the technical admin, a new contract is deployed. Immediately, the address of initiator is restored and a new hash is created for the complete data block.

The contract along with the application is restored in the form of a block and a hash is created. The transaction is sent along with the block for further processing. The address of the applicant and the technical admin is restored in the data block. As soon as the information along with the addresses of the applicant and Ministry of Education representatives is completed, the block is sent to all the universities/educational institutes. A new transaction for verification of the documents is triggered by the Ministry of Education. The student information related to his transcript verification is uploaded in the contract along with the new address.

The next stage is the verification at the University level by the stakeholder responsible for doing this work. The new transaction of the degree record update is done and verified by the stakeholder. A further new copy of the updated contract is deployed along with the existing copy received by the University office. The return address is maintained in the newly created block and further hash is obtained. The hashed block, comprises of the acknowledgement, return address, updated contract value and the address of the previous block. Finally, the University representative updates the information back to the Ministry of education. Further, if the candidate has taken courses across various universities, subsequent information and verification blocks are added in the similar way. After the approval from all the educational activities.

The regulatory authority collaborates to merge all the transactions and upgrade the final contract. The final verified records of the transcript are then submitted to the Ministry of education. This block, comprises of all the agencies involved in the verification and their respective hash values. Once the entire block is completed for the transaction requested by the applicant, the Ministry of Education sends the response back to the blockchain.

The applicant receives back the verified transcript credentials from the system with the help of his login. The next stage counts for sharing this verified transcript to another university or government agency or recruitment office. All the public domain users will be able to verify and acknowledge the final attested copy of the transcript. This final copy will not be able to get edited or modified by any of the users in the public domain. The privacy of the data submitted in the blockchain remains secure and unaltered.

After the final update of the transaction, the data block along with the hash address is pushed on the P2P network across the world. Once the transaction is completed, all the ledger entries are updated in the decentralized database system for use by the blockchain. The hash value ensures that the information is not updated/compromised at any of the node. Any organization seeking the information of a candidate’s transcript, can access the shared transcript of the candidate in the public domain with limited privileges of only accessing the information and not able to updated.

## Experimental Setup and Observations

### Technical deployment

The proposed model in the Transcript Verification Scenario by the Ministry of Education comprises of various technical parts. To understand the model better we have created a prototype for the same model and test it on the local area network of the Shaqra University. The setup of the prototype was deployed on macOS with all the dependencies as well as Smart Contracts. This section briefly describes the entire process for hosting the MOETVBC Application for Transcript Verification as a protype of the model in this study.

• Creation of test network: We create the test network keeping in mind the importance of the security. The test network is based on a Mac machine having M2 Chip and running Monterey Operating System. This machine is acting as a secure server with firewall and security features. The hyperledger need to be installed with some preavailable tools. We installed Homebrew as the package manager to bring the needful packages at the destination/server end. The latest version of GIT was installed along with the Homebrew as a prerequisite for the system. The system also need to have cURL package installed on the macOS to be used in the deployment of the Hyperledger Fabric. The next step was to install Docker and Docker Compose to enable the Fabric Docker image at the destination site. We then installed the SDK application for the blockchain Code called as “Go”. Finally to manage the channel configurations we installed a framework called as JQ. This will handle the channel transactions and manage the communication for smart contracts between two parties.
• Generate organization on the test network: The next step after the installation of the prerequisites and creating the Test Network was to download some fabric samples and docker images to the system. We downloaded the latest version of the Fabric samples from the Git repository. The samples for the Fabric were downloaded from the repository at a location called as hyperledger in the Home directory of the macOS. The cURL was used to download these samples in the directory of the hyperledger. The masterscript for downloading the context as taken from https://raw.githubusercontent.com/hyperledger/fabric/master/scripts/bootstrap.sh. This script comprises of all the samples and channel function required for the creation of needful framework.
• Create a channel to connect these organization: The fabric samples that we have downloaded comprises of the test-network directory. We will create the test networks and channel in this location at the macOS Home directory. A new test network was created and then we run the docker command to pull the docker images fromt he samples that we have downloaded in the previous step. This loads 4 containers with the identifications as tools, orderer and Peer1 and Peer2. These containers are loaded in the memory at hyperledger fabric directory in the macOS. After the successfull creation of the network we created a channel and join this network to the newly created channel.
• Deploy the blockchain code for the smart contract: We created a packaged smart contract in code in the binary library file for its deployment on the network that we have created at the testing site. The blockchain code along with the dependencies were installed with the golang code in the directory. The smart contract package was created with the same technique and wrapped up for use in the home directory of the macOS. This fabric chaincode was zipped with the help of tar utility at the macOS. The nest step was to install the fabric code on the Peer1 role. We committed the installation of the blockchain code package at Peer1 role for the first organization. The similar step were accomplished for the Peer2 as well on the test network. Both the Peer1 and Peer2 for two organizations have to commence and approve the blockchain code. The installation of the code was done and package ID for both the code was taken and assigned to the variables in the local directory of the roles. Once the package ID was completed and commenced then we were able to commit the blockchain code to the channel that was created during the creation of the test network at the site. Finally the code which was deployed on the channel was committed to be used on the channel as an authentic piece of code. Adding items on the blockchain code will thus be helpful now for the smart contract mechanism. Any item whihc is not a trustworthy component shall be discarded and only the sampled items will work as a smart contract package for the Peer1 and Peer2 organization only. This ensure the authenticity and security of the test network.

### Operations of MOETVBC

**Figure 5:**
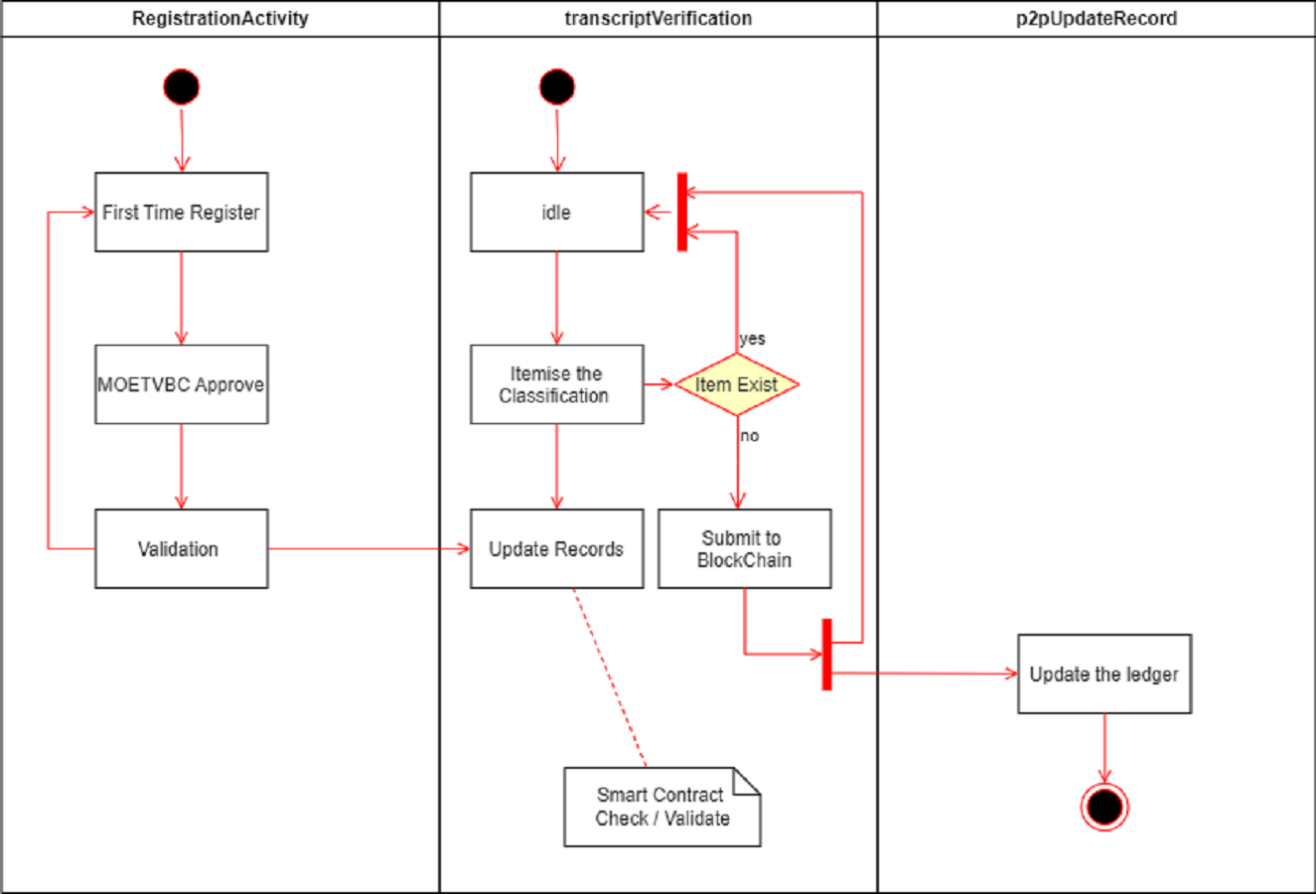
Node transactions in the P2P network environment for MOETVBC application.

Various nodes are supported in the P2P network represented in the model in this study. However The nodal activities that takes place at relevant node is depicted in the Node Transactions Fig. 5 for the MOETVBC Application. The three main nodes are RegistrationActivity, transactionVerification and the p2pUpdateRecord node. The entire procedure which takes place is explained in the Fig. 5 below.

### Working of the model and the activities

**Figure 6:**
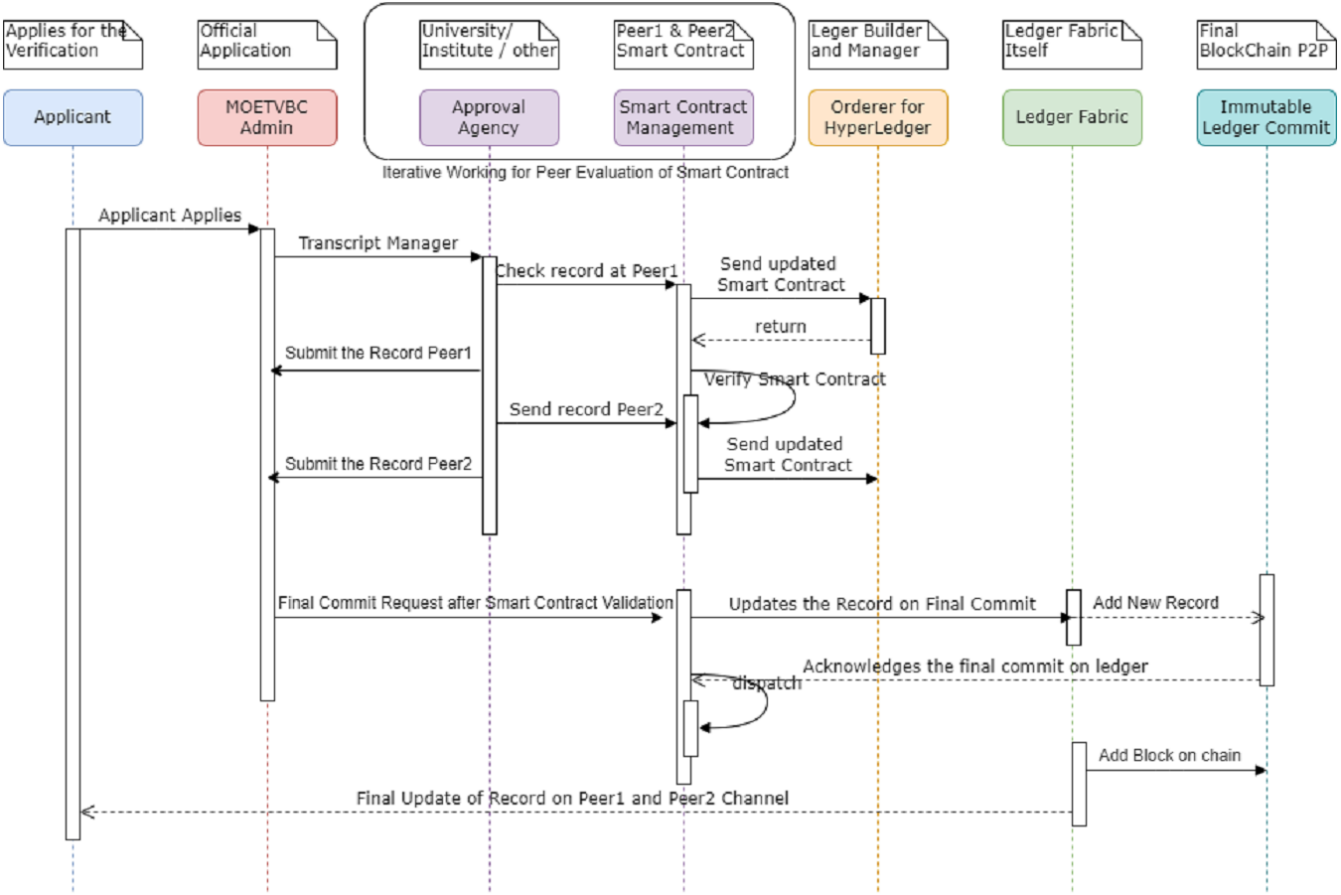
Sequence diagram for MOETVBC application at peer nodes.

The entire model proposed in this study works with the hyperledger and the blockchain code for the transcript verification. The procedure for the peer nodes and the smart contracts of the system is explained in the Sequence Diagram represented in the Fig. 6. The main identifying feature of the proposed blockchain Model is the use of Smart Contract. Peer1 and Peer2 acceptance of the Smart Contract is just not enough in the prescribed model. The eligibility of the model lies in the fact that the smart contract is also accepted by the entire channel in the blockchain.

The acceptance of the Smart Contract by the channel and Peers results in the final acceptance of the Transcript Block being accepted by the Hyperledger. The commit takes place at the hyperledger and is posted across all the nodes in the P2P network. In our case the test network receives the acceptance block for the Peer1 and Peer2 Smart Contract validation. The final nodes are committed on an immutable hyperledger and taken away for a read only block.

**Figure 7:**
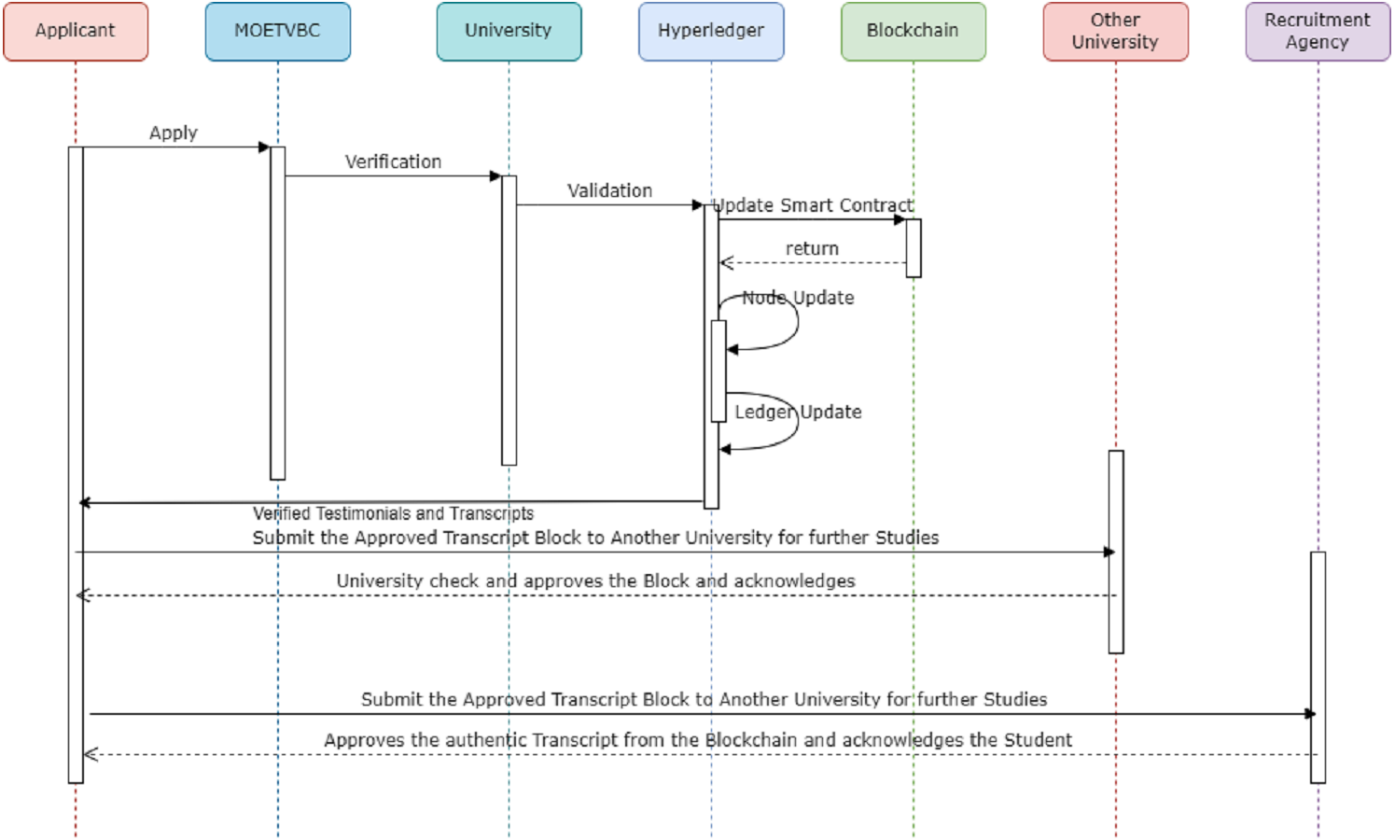
Sequence diagram for MOETVBC transcript verification process for saudi arabian students.

Figure 7 above is a sequence diagram for the MOETVBC Transcript Verification process. The applicant (a student/applicant to any recruitment agency across Saudi Arabia) applies on the MOETVBC for transcript verification. The admin/application requests the university (Peer1) for the approval of the applicants transcripts. The university updates the entity on the block related to the applicant on the hyperledger. The self validation of the smart contract on the channel created for the applicant identity is completed and the nodes on the hyperledger are acknowledged. Finally the blockchain is updated for the use from another peer. As a use case, if the applicant tries to apply to another university/recruitment agency (Peer2) for his future, then the smart contracts is activated for the university on the blockchain. This ensures the Peer2 about the security as well the actual validation of the testimonials for the student. Once the university/recruitment agency or any other stakeholder verifies the data on the blockchain of the MOETVBC, it gets accepted by the organization and the acknowledgement goes back to the sender. In this way the complete prototype works on the hyperledger fabric to ensure the transcript verification process.

### MOETVBC deployment images

**Figure 8:**
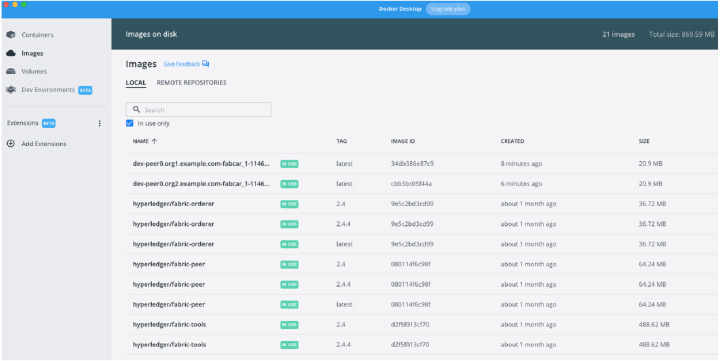
Docker Image Running for Peer1 and Peer2 for two organizations on the macOS.

**Figure 9:**
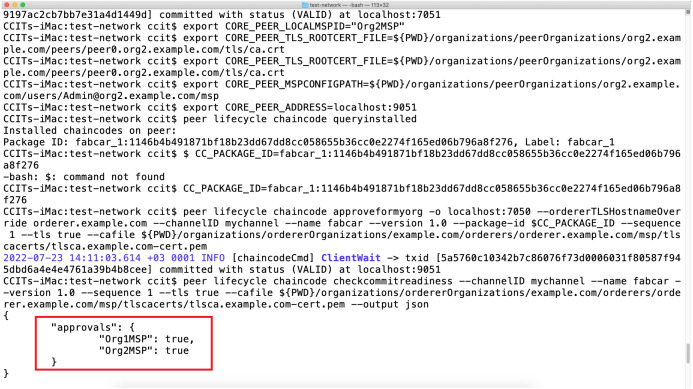
Smart contract acceptance by the two peers on the channel in the blockchain.

Once the smart contracts are accepted by the two peers on the channel defined in the prototype, the further update on the hyperledger is possible in a very easy way. The Figs. 8 and 9 above represents the hyperledger fabric running for the prototype that we have deployed. The docker images for the prototype represents the two Peers running and their respective demonstrative site addresses. While the Fig. 9 represents the actual contract acceptance JSON file for the two peers who are communicating with the blockchain. The acceptance is represented in the red margins which proves the validity of the smart contract and acceptance by the peers.

### Limitations of the model

All the prototypes/proposed models do have some limitations. There are certain shortcomings and challenges which are observed for the proposed model:

• The model that we have proposed is dependent on the inputs given by the Ministry of Education and the policies which are exclusively decided by the regulatory body. The block stockholders on the ledger must assure that all the information which is prescribed by the Ministry should be taken into compliance (Uddin, 2021). In collaboration with the Universities, the education regulatory body must try to provide the mechanism with a transparency and well-defined architecture.
• The architecture proposed in this blockchain coagulates the inputs from various stakeholders. The data supplied by these organizations are in the form of chunks and proper investigation against all the sizes and storage of data remains a challenge. Various federal organizations, institutes, government offices and agencies collaborate for transcript verification. It is expected that the end user must get meaningful educational transaction completed (Khan et al., 2021a). But cross-platform accomplishment for the existing blockchain-based system is expected to be achieved.
• The blockchain is a decentralized and ledger-based mechanism in which the universities along with the Ministry of Education will be able to share valuable inputs for a student’s transcript. With the help of hyper ledger fabric these ledgers are actually updated and information is circulated across all the P2P nodes. The most important task in this entire process is to generate certificates and contracts between various certifying authorities and stakeholders to provide trust between the parties (Khan et al., 2021b). This is still remaining a challenge for various organizations since it consumes more power, cost and time due to the decentralized nature of the storage.

## Conclusion

The article summarizes the key problems in the Transcript Verification Procedure across the Kingdom of Saudi Arabia. A solution is proposed with the help of the blockchain framework, precisely hyperledger fabric. The MOETVBC framework proposed is very consistent in certificate verification as well as it is based on the novel blockchain platform. The ledgers are distributed across global P2P Network nodes and work with smart contracts to provide a robust and secure mechanism for verification of students certificates and degrees. The decentralization of the platform proposed in this research assures the authenticity as well as the integrity of the data in the blocks. The hash code generated and stored in the blocks makes the system very competent against any cyber-attacks or forgery of information. The transparency of the entire proposed framework guarantee forgery proofing and prevents the records from any hazard. The student convenience and educational body regulation are indeed two important factors that are considered during development of this framework. The Ministry of Education can benefit with the platform proposed using blockchain. This will result in the high availability and verification of student certificates and transcript in a very easy and authentic way. A prototype was created and verified for the model proposed in this study. The result is evident that the smart contracts are a very great means to ensure the authenticity and security in the blockchain. Two peers can establish a good communication and work really well when it comes to the blockchain. The complete working of the prototype is explained in the form of sequence diagrams and the experimental setup is also provided in the previous section. This prototype of the proposed model ensures that this application will be really useful for the students of the Saudi Arabian universities/institutes to use their validated credentials of transcripts for various purposes like further studies or recruitment. Thus the MOETVBC can act as a great source for blockchain based Transcript Verification for Saudi Arabia.

## Supplemental Information

10.7717/peerj-cs.1120/supp-1 Supplemental Information 1 Smart Contract exchange dataset between Peer Entities as well as organization to combine the blockchain exchange of data

10.7717/peerj-cs.1120/supp-2 Supplemental Information 2 Terminal bash script to generate the Smart Contracts between the Peer 1 and Peer 2 in the Blockchain

## Additional Information and Declarations

### Data Availability

The following information was supplied regarding data availability: The raw data is available in the Supplemental File. The bash files are the shell scripts that run directly on Linux or macOS based systems. Use CYGWIN (an open source tool to enable running Linux based distributions and applications on Windows environment) to run these files on Windows. CYGWIN is available at: https://www.cygwin.com/setup-x86_64.exe. Installation instructions are available at: https://www.cygwin.com/install.html.
